# Lifestyle and Anthropometric Predictors of Hypertension Among Adults Attending Debark General Hospital, Northwest Ethiopia: An Unmatched Case–Control Study

**DOI:** 10.1155/ijhy/5543891

**Published:** 2026-02-08

**Authors:** Kaleab Tesfaye Tegegne, Eleni Tesfaye Tegegne, Mekibib Kassa Tessema, Samuel Ermiyas Teshome, Aemero Asmamaw Chalachew, Tadele Kassahun Wudu, Asmamaw Zegeye Workneh, Moges Tadesse Abebe, Jenberu Mekurianew Kelkay, Derebe Marie Adugna

**Affiliations:** ^1^ Department of Public Health, College of Health Science, Debark University, Debark, Ethiopia, dku.edu.et; ^2^ Department of Nursing, School of Nursing, College of Medicine and Health Sciences, University of Gondar, Gondar, Ethiopia, uog.edu.et; ^3^ Leishmania Research and Treatment Center, University of Gondar, Gondar, Ethiopia, uog.edu.et; ^4^ Department of Natural Resource Management, College of Agriculture, Debark University, Debark, Ethiopia, dku.edu.et; ^5^ Department of Educational Psychology, Dean of the College of Social Sciences and Humanities, University of Gondar, Gondar, Ethiopia, uog.edu.et; ^6^ Department of Statistics, College of Natural and Computational Science, Debark University, Debark, Ethiopia, dku.edu.et; ^7^ Department of Mathematics, President of Debark University, Debark, Ethiopia; ^8^ Department of Pediatric and Child Health Nursing, College of Health Science, Debark University, Debark, Ethiopia, dku.edu.et; ^9^ Department of Agricultural Economics, College of Agriculture and Environmental Conservation, Debark University, Debark, Ethiopia, dku.edu.et

**Keywords:** dietary salt, Ethiopia, hypertension, lifestyle factors, obesity, physical activity, predictors

## Abstract

**Background:**

Hypertension is a growing public health concern in Ethiopia, contributing substantially to cardiovascular morbidity and mortality. Identifying predictors of hypertension is crucial for effective prevention and control.

**Objective:**

To identify the lifestyle and anthropometric predictors of hypertension among adults attending Debark General Hospital, Northwest Ethiopia.

**Methods:**

An unmatched case–control study was conducted from January to March 2025, including 640 participants (128 hypertensive cases and 512 normotensive controls) with a 1:4 case‐to‐control ratio. Cases were adults diagnosed with hypertension (SBP ≥ 140 mmHg and/or DBP ≥ 90 mmHg or on antihypertensive treatment). Controls were normotensive adults attending the hospital for other health issues. Data were collected using a structured questionnaire and anthropometric measurements. Multivariable logistic regression was performed using Stata to identify independent predictors of hypertension.

**Results:**

The multivariable analysis identified age ≥ 45 years (AOR = 3.62; 95% CI: 2.11–6.20), obesity (BMI ≥ 30 kg/m^2^) (AOR = 2.95; 95% CI: 1.78–4.89), low physical activity (AOR = 2.47; 95% CI: 1.45–4.19), high dietary salt intake (AOR = 2.33; 95% CI: 1.32–4.11), family history of hypertension (AOR = 3.14; 95% CI: 1.89–5.22), alcohol consumption (AOR = 2.01; 95% CI: 1.17–3.44), and low fruit intake (< 5 servings/week) (AOR = 1.89; 95% CI: 1.08–3.29) as significant predictors of hypertension (all *p* < 0.05).

**Conclusion:**

The study identified key modifiable predictors of hypertension, including obesity, physical inactivity, high salt intake, alcohol use, and low fruit consumption, along with nonmodifiable factors such as older age and family history among adults in Northwest Ethiopia. These findings highlight the need for integrated preventive interventions targeting lifestyle modification and early screening in the Ethiopian healthcare system.

## 1. Introduction

Hypertension is a leading cause of cardiovascular diseases, stroke, kidney failure, and premature mortality, making it one of the most important global public health challenges [1]. The World Health Organization reports that hypertension is a major cause of premature death worldwide, affecting an estimated 1.28 billion adults, with the majority living in low‐ and middle‐income countries [2]. In these settings, rapid urbanization, dietary transitions, and sedentary lifestyles have fueled a steady rise in hypertension, contributing substantially to the burden of noncommunicable diseases (NCDs) and straining already resource‐limited health systems [3]. Because hypertension is often asymptomatic in its early stages, it frequently remains undiagnosed and untreated, increasing the risk of life‐threatening complications.

In Ethiopia, hypertension prevalence has been rising over the past 2 decades, reflecting the country’s epidemiological transition. National estimates suggest that about one in five adults is hypertensive [4], with studies reporting prevalence ranging from 15% to over 30% depending on the setting [5, 6]. This upward trend has been linked to behavioral and sociodemographic changes, including increased consumption of high‐salt processed foods, reduced physical activity, alcohol use, and population aging [7]. Although urban areas report higher prevalence, rural and semiurban communities are increasingly affected as urban lifestyles spread. Alarmingly, awareness, treatment, and control rates remain very low in Ethiopia, with less than 40% of hypertensive individuals being diagnosed and only a fraction adequately controlled [8]. This gap poses a serious risk of escalating cardiovascular morbidity and mortality.

Several studies in Ethiopia have identified predictors of hypertension, such as advanced age, obesity, physical inactivity, high dietary salt intake, alcohol consumption, and family history [5–7]. However, these findings are often context‐specific, with results varying across regions due to differences in lifestyle, cultural practices, and healthcare access. For example, some studies report a strong association between khat chewing and hypertension [9], whereas others find no significant relationship [10]. Such inconsistencies highlight the need for localized evidence to better inform prevention and control efforts.

Despite the growing burden of hypertension in Ethiopia, little is known about its predictors in Northwest Ethiopia, particularly in underserved areas such as Debark. This region is experiencing epidemiological and lifestyle changes but has limited healthcare infrastructure and preventive services. The absence of localized data hampers the design of targeted interventions and equitable healthcare planning. Without such evidence, hypertension will likely remain underdiagnosed and poorly controlled, leading to preventable complications and increased healthcare costs.

To address this gap, the present study was conducted (mark) to identify sociodemographic, behavioral, and anthropometric predictors of hypertension among adult patients attending Debark General Hospital.

Unlike previous studies, this research provides context‐specific evidence from a semiurban and rural setting, thereby contributing novel insights into the determinants of hypertension in an understudied population [11]. The findings are expected to guide targeted prevention strategies, inform clinical practice, and support public health programs aligned with the World Health Organization’s Global Action Plan for the prevention and control of NCDs [12].

## 2. Methods

### 2.1. Study Design and Setting

An institution‐based unmatched case–control study was conducted at Debark General Hospital, Northwest Ethiopia, from January to March 2025. The hospital is located in Debark town, which lies approximately 830 km from Addis Ababa and serves as the primary referral center for the North Gondar Zone of the Amhara Regional State. Debark district has an estimated population of over 212,000, with most residents engaged in mixed farming and small‐scale trade (Ethiopian Statistics Service Population Projection). The staple foods in the area include barley, wheat, maize, and potatoes, which are widely consumed. The hospital provides outpatient and inpatient services for communicable diseases and NCDs, including chronic disease management such as hypertension and diabetes clinics.

### 2.2. Source Population

The source population included all adult patients (≥ 18 years) attending Debark General Hospital during the study period.

### 2.3. Study Population

The study population consisted of

• Cases: Adult patients diagnosed with hypertension (systolic blood pressure ≥ 140 mmHg and/or diastolic blood pressure ≥ 90 mmHg or on antihypertensive medication), confirmed by physician diagnosis and current measurement.
• Controls: Normotensive adults with systolic < 130 mmHg and diastolic < 85 mmHg, with no history of hypertension or prehypertension), recruited from patients visiting for unrelated health concerns.
• Eligibility Criteria
   Inclusion Criteria: Adults aged 18 years and above attending the hospital during the study period. Cases were those with confirmed hypertension; controls were those without hypertension, verified by medical records and blood pressure measurement.
   Exclusion Criteria: Pregnant women (due to physiological changes in blood pressure), critically ill patients unable to respond, and individuals with incomplete or unreliable medical records.
   Justification for Age and Control Definitions: The adult age threshold (≥ 18 years) was used as it is the legal age of adulthood in Ethiopia and aligns with the operational definition used in the national STEPS survey for NCD risk factors. The control group was defined with a stricter blood pressure cutoff (< 130/85 mmHg) to exclude individuals with prehypertension, thereby reducing misclassification bias and providing a clearer comparison against true hypertensive cases.

#### 2.3.1. Sample Size Determination

The sample size was calculated using the double population proportion formula in Epi Info version 7.2. Parameters included 95% confidence level, 80% power, a 1:4 case‐to‐control ratio, 20% prevalence of alcohol consumption among controls, and an assumed odds ratio of 2 for alcohol consumption [12]. Adding a 10% allowance for potential nonresponse yielded a final sample size of 640 participants (128 cases and 512 controls).

#### 2.3.2. Sampling Procedure

Cases were recruited consecutively from the chronic disease clinic and medical inpatient ward until the required sample size was reached. Controls were selected systematically from patients attending outpatient departments (OPDs) for nonhypertension‐related services (e.g., minor injuries and infections). To ensure comparability, controls were frequency‐matched to cases by age group (in decades). Consecutive sampling ensured all eligible cases during the study period were included.

#### 2.3.3. Data Collection Tools and Procedures

Data were collected using a structured, interviewer‐administered questionnaire adapted from the WHO STEPwise Approach to NCD Surveillance (STEPS) and relevant literature [13, 14]. The questionnaire captured the following.

#### 2.3.4. Sociodemographic Characteristics

• Behavioral factors (dietary practices, physical activity, and alcohol and tobacco use)
• Medical history (family history of hypertension and comorbidities)
• Anthropometric measurements (height, weight, and BMI)

Measurement procedures included the following:

• Blood Pressure: Measured using a calibrated digital sphygmomanometer following WHO guidelines [15]. Three readings were taken at 5‐min intervals; the average of the last two was used.
• Weight and Height: Measured using a calibrated scale and stadiometer with participants wearing light clothing and no shoes.
• BMI: Calculated as weight (kg)/height (m^2^).
• Salt Intake and Fruit/Vegetable Consumption: Assessed via self‐reported frequency and converted to WHO cutoffs [16, 17].
• Physical Activity: Measured using the WHO Global Physical Activity Questionnaire (GPAQ) [18].

### 2.4. Operational Definitions

• Hypertension (Case): SBP ≥ 140 mmHg and/or DBP ≥ 90 mmHg, or use of antihypertensive medication [15].
• Normotensive (Control): SBP < 130 mmHg and DBP < 85 mmHg, with no history of hypertension or prehypertension [15].
• Obesity: BMI ≥ 30 kg/m^2^ [16].
• Normal Weight: BMI < 25 kg/m^2^ [16].
• Low Physical Activity: < 150 min of moderate‐intensity or < 75 min of vigorous‐intensity activity/week [18].
• High Dietary Salt Intake: > 5 g/day [16].
• Low Fruit Intake: < 400 g (or < 5 servings of 80 g) per day, based on WHO healthy diet recommendations [17].
• Adequate Fruit Intake: ≥ 5 servings/week (reference) [17].
• Family History of Hypertension: At least one first‐degree relative (parent/sibling) with hypertension.
• Alcohol Consumption: Intake of alcohol ≥ 1 time per week in the past 6 months [19].
• Dietary exposures were assessed through a structured questionnaire. High salt intake was defined according to WHO guidelines as > 5 g/day. Because direct measurement was not feasible, participants were asked about the frequency of adding extra salt to meals and the consumption of salty foods. Interviewers provided practical explanations [20].

### 2.5. Data Quality Assurance

The questionnaire was translated into Amharic and back‐translated to English to maintain semantic accuracy. Six trained health professionals collected data under the supervision of two public health experts. Training covered study objectives, ethical procedures, interview techniques, and standard measurement protocols. The questionnaire was pretested on 5% of the sample in a neighboring facility. Daily supervision, rechecking of completed forms, and periodic recalibration of equipment enhanced reliability and validity.

### 2.6. Data Processing and Analysis

Data were entered into EpiData version 4.6 and exported to Stata version 16 for cleaning and analysis. Descriptive statistics such as frequencies, means, and standard deviations were used to summarize variables. Bivariable logistic regression was first performed to assess the crude association between each independent variable and hypertension. Variables with *p* < 0.25 in the bivariable analysis were entered into a multivariable logistic regression model to control for potential confounders. Adjusted odds ratios (AORs) with 95% confidence intervals (CIs) were reported, and *p*‐values < 0.05 were considered statistically significant. The Hosmer–Lemeshow goodness‐of‐fit test was used to assess model fitness, and multicollinearity was checked using variance inflation factors (VIFs).

### 2.7. Ethical Considerations

Ethical approval for this study was obtained from the Institutional Review Board (IRB) of Debark University (Reference No. IRB/DU/102/2024, dated December 15, 2024). Additionally, a formal permission letter was secured from the Debark General Hospital administration. Before data collection, written informed consent was obtained from each participant after clearly explaining the objectives, potential risks, and benefits of the study. Confidentiality and privacy were strictly maintained by using anonymous codes, and participants were informed of their right to withdraw at any stage without any repercussions. This study was conducted in accordance with the principles outlined in the Declaration of Helsinki.

## 3. Result

Table 1 presents the sociodemographic, behavioral, and clinical characteristics of the 640 study participants (128 cases and 512 controls) at Debark General Hospital. Age distribution shows a higher proportion of cases in the older adult category (≥ 60 years, 42.2%). A higher proportion of hypertension cases were aged ≥ 45 years (64.8%) and obese (41.4%) compared to controls. Low physical activity, high salt intake, family history of hypertension, alcohol consumption, and low fruit intake were also more common among cases.

**Table 1:** Sociodemographic, behavioral, and clinical characteristics of study participants, Debark General Hospital, 2025.

Variable	Category	Cases (*n* = 128) *n* (%)	Controls (*n* = 512) *n* (%)	Total (*n* = 640) *n* (%)
Age group	18–24 years	12 (9.4)	142 (27.7)	154 (24.1)
	25–59 years	62 (48.4)	328 (64.1)	390 (60.9)
	< 60 years	54 (42.2)	42 (8.2)	96 (15.0)

BMI	Normal (< 25 kg/m^2^)	53 (41.4)	326 (63.7)	379 (58.5)
	Overweight (25–29.9 kg/m^2^)	22 (17.2)	99 (19.3)	121 (19.2)
	Obese (≥ 30 kg/m^2^)	53 (41.4)	87 (17.0)	140 (22.3)

Physical activity	Sufficient	56 (43.8)	338 (66.0)	394 (61.6)
	Low	72 (56.2)	174 (34.0)	246 (38.4)

Dietary salt intake	Low	43 (33.6)	337 (65.8)	380 (59.1)
	High	85 (66.4)	175 (34.2)	260 (40.9)

Family history of hypertension	No	75 (58.6)	368 (71.9)	443 (68.8)
	Yes	53 (41.4)	144 (28.1)	197 (31.2)

Alcohol intake	No	83 (64.8)	364 (71.1)	447 (69.9)
	Yes	45 (35.2)	148 (28.9)	193 (30.1)

Fruit intake	Adequate (≥ 5 servings/week)	77 (60.2)	343 (67.0)	420 (65.5)
	Low (< 5 servings/week)	51 (39.8)	169 (33.0)	220 (34.5)

Marital status	Married	93 (72.7)	358 (69.9)	451 (70.5)
	Single/widowed/divorced	35 (27.3)	154 (30.1)	189 (29.5)

Gender	Male	76 (59.4)	250 (48.8)	326 (50.9)
	Female	52 (40.6)	262 (51.2)	314 (49.1)

Occupation	Government/private employee	49 (38.3)	153 (29.9)	202 (31.6)
	Farmer	27 (21.1)	120 (23.4)	147 (23.0)
	Merchant	30 (23.4)	106 (20.7)	136 (21.3)
	Unemployed/housewife/others	22 (17.2)	133 (26.0)	155 (24.2)

Residence	Urban	69 (53.9)	267 (52.1)	336 (52.5)
	Rural	59 (46.1)	245 (47.9)	304 (47.5)

Religion	Orthodox Christian	111 (86.7)	432 (84.4)	543 (84.8)
	Muslim/other	17 (13.3)	80 (15.6)	97 (15.2)

Monthly income (ETB)	< 2000 ETB	41 (32.0)	163 (31.8)	204 (31.9)
	2000–4999 ETB	56 (43.8)	227 (44.3)	283 (44.2)
	≥ 5000 ETB	31 (24.2)	122 (23.8)	153 (23.9)

### 3.1. Bivariable and Multivariable Logistic Regression Analysis

Table 2 presents the bivariable and multivariable logistic regression analyses. In the bivariable analysis, age ≥ 60 years, obesity, low physical activity, high salt intake, family history of hypertension, alcohol consumption, and low fruit intake showed significant crude associations with hypertension (*p* < 0.05).

**Table 2:** Bivariable and multivariable logistic regression analysis of predictors of hypertension (*n* = 640).

Variable	COR (95% CI)	*p*‐Value	AOR (95% CI)	*p*‐Value
Age group				
≥ 45 years	4.25 (2.70–6.68)	< 0.001^∗∗∗^	3.62 (2.11–6.20)	< 0.001^∗∗∗^
18–44 years (Ref)	—	—	—	—
Body mass index (BMI)				
Obese (≥ 30 kg/m^2^)	3.45 (2.15–5.55)	< 0.001^∗∗∗^	2.95 (1.78–4.89)	< 0.001^∗∗∗^
Normal (< 25 kg/m^2^) (Ref)	—	—	—	—
Physical activity				
Low	2.88 (1.81–4.58)	< 0.001^∗∗∗^	2.47 (1.45–4.19)	0.001^∗∗^
Sufficient (Ref)	—	—	—	—
Dietary salt intake				
High intake	2.66 (1.65–4.28)	< 0.001^∗∗∗^	2.33 (1.32–4.11)	0.003^∗∗^
Low intake (Ref)	—	—	—	—
Family history of hypertension				
Yes	3.72 (2.37–5.83)	< 0.001^∗∗∗^	3.14 (1.89–5.22)	< 0.001^∗∗∗^
No (Ref)	—	—	—	—
Alcohol consumption				
Yes	2.23 (1.40–3.56)	0.001^∗∗^	2.01 (1.17–3.44)	0.011^∗^
No (Ref)	—	—	—	—
Fruit intake				
Low (< 5 servings/week)	2.12 (1.28–3.50)	0.004^∗∗^	1.89 (1.08–3.29)	0.025^∗^
Adequate (Ref)	—	—	—	—
Gender				
Female	1.18 (0.79–1.77)	0.13	1.12 (0.71–1.76)	0.619
Male (Ref)	—	—	—	—
Marital status				
Married	1.24 (0.78–1.96)	0.16	1.08 (0.63–1.86)	0.777
Unmarried (Ref)	—	—	—	—
Residence				
Urban	0.88 (0.59–1.31)	0.19	0.92 (0.58–1.48)	0.733
Rural (Ref)	—	—	—	—
Religion				
Orthodox	1.16 (0.69–1.95)	0.21	1.02 (0.58–1.82)	0.953
Other (Ref)	—	—	—	—
Occupation				
Government employee	1.41 (0.84–2.38)	0.196	1.25 (0.70–2.23)	0.453
Merchant	1.38 (0.78–2.44)	0.205	1.13 (0.61–2.10)	0.690
Housewife	1.17 (0.68–2.01)	0.203	1.05 (0.56–1.96)	0.878
Farmer (Ref)	—	—	—	—
Monthly income				
≥ 3000 ETB	1.10 (0.72–1.70)	0.11	0.97 (0.59–1.59)	0.907
< 3000 ETB (Ref)	—	—	—	—

Abbreviations: AOR, adjusted odds ratio; COR, crude odds ratio.

^∗^ *p* < 0.05,  ^∗∗^ *p* < 0.01, and  ^∗∗∗^ *p* < 0.001.

In the final adjusted model, seven factors remained significant independent predictors of hypertension. Participants aged ≥ 45 years had higher odds of hypertension compared to those aged 18–44 years (AOR = 3.62; 95% CI: 2.11–6.20). Other significant predictors included obesity (AOR = 2.95), low physical activity (AOR = 2.47), high dietary salt intake (AOR = 2.33), family history of hypertension (AOR = 3.14), alcohol consumption (AOR = 2.01), and low fruit intake (AOR = 1.89).

## 4. Discussion

The findings of this study indicate that age ≥ 45 years, obesity, low physical activity, high dietary salt intake, family history of hypertension, alcohol consumption, and low fruit intake were independently associated with hypertension in the multivariable analysis. Although the AORs for most predictors were lower than the crude odds ratios, they remained statistically significant, indicating independent associations after controlling for confounders.

The findings are consistent with previous evidence from Ethiopia and other low‐ and middle‐income countries, confirming that both modifiable lifestyle factors and nonmodifiable characteristics contribute to the rising hypertension burden. In particular, the strong association with older age and obesity aligns with global epidemiological patterns, as vascular changes and excess adiposity are well‐documented drivers of elevated blood pressure [21–24]. The significance of behavioral risk factors such as alcohol use, inadequate fruit intake, physical inactivity, and high salt consumption underscores the role of lifestyle transitions in semiurban Ethiopian communities, where dietary and activity patterns increasingly mirror urban lifestyles [5, 6, 25–34].

One notable finding is the role of low fruit intake as a predictor of hypertension in this setting. Although international evidence has long supported the protective effects of fruits, this determinant is less frequently emphasized in local studies. In the Ethiopian context, this may reflect limited affordability and accessibility of fruits, suggesting that nutrition‐sensitive interventions could have a meaningful impact. Similarly, the high prevalence of alcohol consumption and its strong association with hypertension emphasize the need for culturally sensitive prevention strategies, as alcohol is both socially accepted and economically accessible in the region. Our multivariable model shows that these factors (e.g., obesity, salt, and activity) act as independent, additive predictors. Although biological interactions (e.g., salt sensitivity exacerbated by obesity) are plausible, our analysis did not detect significant statistical interaction terms among the main variables, suggesting their effects are largely separate within this study’s power.

These results are consistent with previous studies in Ethiopia and other low‐income settings, which have reported similar predictors of hypertension [35, 36]. The strong association with older age and obesity aligns with global evidence on vascular aging and adiposity‐related mechanisms [20, 37, 38].

These results carry important implications. First, they reaffirm that hypertension prevention in Ethiopia should focus on lifestyle modification particularly reducing salt intake, promoting physical activity, and encouraging healthy dietary practices. Second, they highlight the importance of strengthening primary healthcare services to incorporate routine screening and targeted health education, especially for individuals with a family history of hypertension who are at heightened risk. Finally, they provide locally specific evidence for policymakers, addressing the gap in context‐sensitive data from underserved areas such as Debark.

The role of behavioral factors such as alcohol use, inadequate fruit intake, and physical inactivity underscores the impact of lifestyle transitions in semiurban Ethiopia [39–42].

The study has some limitations, including its case–control design, potential recall bias, and hospital‐based sampling, which may limit generalizability. However, the use of standardized tools and a relatively large sample size strengthens the validity of the findings.

## 5. Conclusion

This study identified both modifiable and nonmodifiable predictors of hypertension among adults in Northwest Ethiopia. Modifiable factors included obesity, physical inactivity, high salt intake, alcohol use, and low fruit consumption, whereas nonmodifiable factors included older age and family history of hypertension. These findings underscore the need for targeted public health interventions focusing on lifestyle modification, community education, and early screening. Future research should include community‐based longitudinal studies to establish causality and evaluate the effectiveness of tailored intervention programs.

## Author Contributions

Kaleab Tesfaye Tegegne conceived and designed the study, coordinated the data collection, performed the statistical analysis, and drafted the manuscript. Eleni Tesfaye Tegegne and Mekibib Kassa Tessema contributed to the study design, supervised the data collection, and critically reviewed the manuscript. Samuel Ermiyas Teshome and Aemero Asmamaw Chalachew provided technical support on data management and interpretation of findings. Tadele Kassahun Wudu assisted with the statistical analysis and data interpretation. Asmamaw Zegeye Workneh and Moges Tadesse Abebe contributed to the manuscript revision and provided expert guidance on public health and clinical aspects. Jenberu Mekurianew Kelkay participated in the literature review and manuscript editing. Derebe Marie Adugna also supported the development of the data collection tools and assisted in manuscript revisions. Correspondence and requests for materials should be addressed to Kaleab Tesfaye Tegegne.

## Funding

This study did not receive any specific grant or financial support from funding agencies in the public, commercial, or not‐for‐profit sectors.

## Disclosure

All authors read and approved the final manuscript.

## Ethics Statement

Ethical approval for this study was obtained from the Institutional Review Board (IRB) of Debark University (Reference No. IRB/DU/102/2024, dated December 15, 2024). Additionally, a formal permission letter was secured from the Debark General Hospital administration. Before data collection, written informed consent was obtained from each participant after clearly explaining the objectives, potential risks, and benefits of the study. Confidentiality and privacy were strictly maintained by using anonymous codes, and participants were informed of their right to withdraw at any stage without any repercussions. This study was conducted in accordance with the principles outlined in the Declaration of Helsinki.

## Consent

The authors have nothing to report.

## Conflicts of Interest

The authors declare no conflicts of interest.

## Data Availability

The datasets generated and/or analyzed during the current study are available from the corresponding author on reasonable request.
